# Evaluating the Dermatological Benefits of Snowberry (*Symphoricarpos albus*): A Comparative Analysis of Extracts and Fermented Products from Different Plant Parts

**DOI:** 10.3390/ijms25179660

**Published:** 2024-09-06

**Authors:** Chanwoo Lee, Hana Cho, Myunsoo Kim, Boae Kim, Young-Pyo Jang, Junseong Park

**Affiliations:** 1 College of Pharmacy, Kyung Hee University, Seoul 02447, Republic of Korea; bryan@miglim.com; 2 Miglim Co., Ltd., A-1309, 30, Songdomirae-ro, Yeonsu-gu, Incheon 21990, Republic of Korea; 3 Technology R&D Institute, ICBIO, 1 Naeyuri 1-gil, Ipjang-meyon, Seobuk-gu, Cheonan-si 31027, Republic of Korea; hana@icbio.co.kr (H.C.); mskim@icbio.co.kr (M.K.); 4 Department of Cosmetic Engineering, Collage of Technology Sciences, Mokwon University, Daejeon 35349, Republic of Korea; kba@mokwon.ac.kr; 5 Department of Engineering Chemistry, Chungbuk National University, Cheongju 28644, Republic of Korea

**Keywords:** *Symphoricarpos albus*, snowberry, *Lactobacillus plantarum*, moisturising, anti-ageing

## Abstract

Skin ageing is influenced by both intrinsic and extrinsic factors, with excessive ultraviolet (UV) exposure being a significant contributor. Such exposure can lead to moisture loss, sagging, increased wrinkling, and decreased skin elasticity. Prolonged UV exposure negatively impacts the extracellular matrix by reducing collagen, hyaluronic acid, and aquaporin 3 (AQP-3) levels. Fermentation, which involves microorganisms, can produce and transform beneficial substances for human health. Natural product fermentation using lactic acid bacteria have demonstrated antioxidant, anti-inflammatory, antibacterial, whitening, and anti-wrinkle properties. Snowberry, traditionally used as an antiemetic, purgative, and anti-inflammatory agent, is now also used as an immune stimulant and for treating digestive disorders and colds. However, research on the skin benefits of Fermented Snowberry Extracts remains limited. Thus, we aimed to evaluate the skin benefits of snowberry by investigating its moisturising and anti-wrinkle effects, comparing extracts from different parts of the snowberry plant with those subjected to fermentation using *Lactobacillus plantarum*. Chlorophyll-free extracts were prepared from various parts of the snowberry plant, and ferments were created using *Lactobacillus plantarum*. The extracts and ferments were analysed using high-performance liquid chromatography (HPLC) to determine and compare their chemical compositions. Moisturising and anti-ageing tests were conducted to assess the efficacy of the extracts and ferments on the skin. The gallic acid content remained unchanged across all parts of the snowberry before and after fermentation. However, Fermented Snowberry Leaf Extracts exhibited a slight decrease in chlorogenic acid content but a significant increase in ferulic acid content. The Fermented Snowberry Fruit Extract demonstrated increased chlorogenic acid and a notable rise in ferulic acid compared to its non-fermented counterpart. Skin efficacy tests revealed that Fermented Snowberry Leaf and Fruit Extracts enhanced the expression of AQP-3, HAS-3, and COL1A1. These extracts exhibited distinct phenolic component profiles, indicating potential skin benefits such as improved moisture retention and protection against ageing. These findings suggest that Fermented Snowberry Extracts could be developed into effective skincare products, providing a natural alternative for enhancing skin hydration and reducing signs of ageing.

## 1. Introduction

The skin performs multiple physiological functions, including protection against UV rays, mitigation of disease risks such as bacteria and toxin infiltration, regulation of body temperature, and preservation of body water retention [1,2]. Over time, skin ageing is influenced by external factors, notably excessive UV exposure, leading to moisture loss, sagging, increased wrinkling, and reduced elasticity [3,4]. Excessive UV radiation impairs the function of the extracellular matrix, resulting in decreased levels of collagen, hyaluronic acid (HA), and aquaporin 3 (AQP-3) [5,6].

Collagen, distributed throughout the dermis, is crucial for maintaining skin elasticity. Reduced collagen levels lead to diminished elasticity and wrinkle formation. HA, a component of the extracellular matrix, contributes to water retention in tissues, the storage and diffusion of cell growth factors and nutrients, and immune system regulation [7,8,9]. HA levels in the skin are regulated by hyaluronic acid synthase (HAS) for synthesis and hyaluronidase (HYAL) for degradation [10]. A reduction in HA is directly associated with increased wrinkling, decreased elasticity, and lower skin water content due to defects in the moisture barrier, leading to epidermal atrophy [7,8,9]. Aquaporins (AQPs) in the epidermis are hydrophobic membrane-embedded proteins that act as transmembrane proteins to transport water from the outside into the cell [11]. Reduced AQP-3 expression is known to cause dry skin, as demonstrated by AQP3 knockout mice exhibiting dry skin and impaired wound healing [12]. 

Fermentation, a biotechnology process, involves the metabolic action of microorganisms to produce and bioconvert various health-beneficial substances [13,14]. Recent advancements in biotechnology have led to various studies on the fermentation of natural products by lactic acid bacteria, exhibiting antioxidant, anti-inflammatory, antibacterial, whitening, and anti-wrinkle properties [15,16,17]. Among lactic acid bacteria, probiotic strains such as *Bifidobacterium*, *Saccharomyces*, *Enterococcus*, *Bacillus*, and *Lactobacillus* have shown benefits for skin health [18,19]. Specifically, *Lactobacillus plantarum* has been documented to exhibit immunomodulatory effects, potentially influencing atopic dermatitis symptoms in adults [20]. 

*Symphoricarpos albus*, commonly known as snowberry, is a deciduous shrub in the family *Caprifoliaceae*, with approximately 15 species, primarily indigenous to North and Central America, with the remaining species originating from western China. Snowberry, also known as waxberry or ghostberry, has flowers approximately 5 mm in length that bloom from May through summer, with berries persisting on branches through winter. The berries, 1–2 cm in diameter, vary in colour from white (common snowberry, *Symphoricarpos albus*) to pink (little leaf snowberry, *Symphoricarpos microphyllus*), blackish purple (Chinese Coralberry, *Symphoricarpos sinensis*), and red (coralberry, *Symphoricarpos orbiculatus*). Other species include Mexican snowberries, creeping snowberries, western snowberries, mountain snowberries, and roundleaf snowberries. Phytochemical studies have identified flavonoids such as quercetin, apigenin, and luteolin, as well as coumarins, aesculin, fraxetin, tannins, iridoids, saponins, triterpenes, sugars, pectins, isoquinoline alkaloids, and choline in snowberries [21]. These berries also contain chlorogenic, quinic, aminobutyric, malic, tartaric, and citric acids. Traditionally, snowberries have been used as antiemetic, purgative, and anti-inflammatory agents, and currently for digestive disorders, colds, and immune stimulation [21]. 

Although phytochemical studies have identified various compounds in snowberries, there is a notable gap in the research regarding the skin benefits of snowberries, particularly concerning the effects of different plant parts and the impact of fermentation. To our knowledge, this is the first study to investigate the skin-enhancing properties of Snowberry Extracts, both in their natural and fermented forms, thereby contributing novel insights to the field of dermatological research.

This study aimed to evaluate the skin benefits of snowberries through fermentation. It compared the moisturising and anti-wrinkle effects of extracts from different parts of the snowberry plant (Snowberry Extract, SE) with extracts fermented using *Lactobacillus plantarum* (Fermented Snowberry Extract, FSE).

## 2. Results

### 2.1. Phytochemical Analysis of SE and FSE

**Figure 1:**
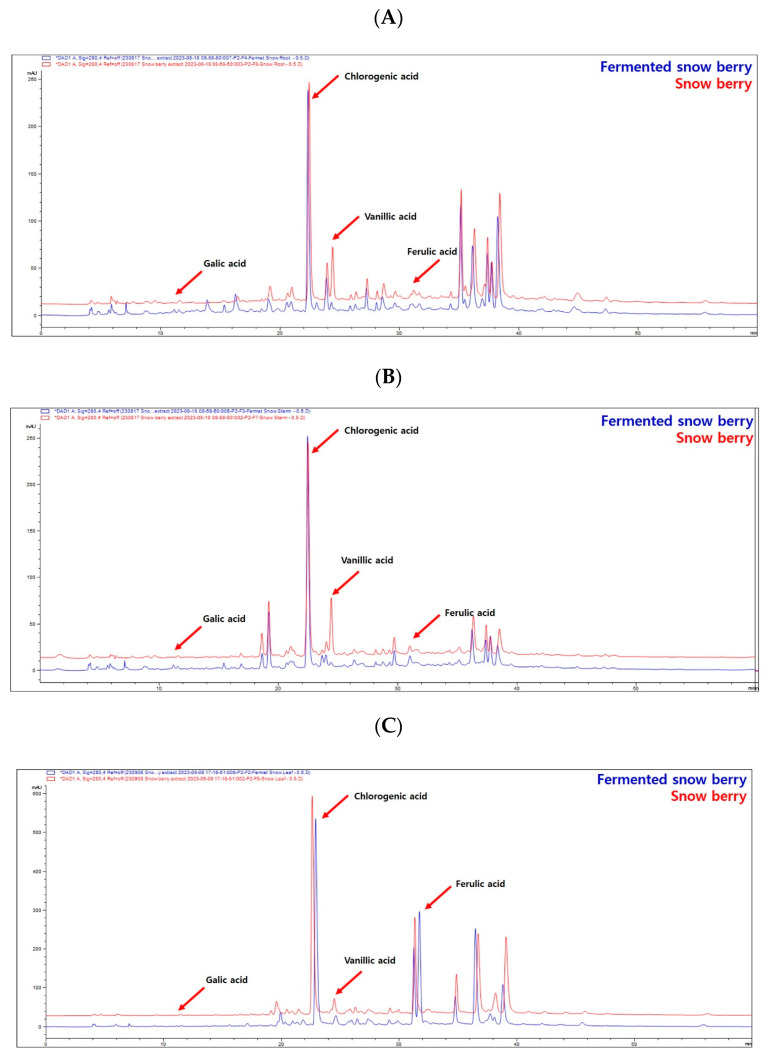

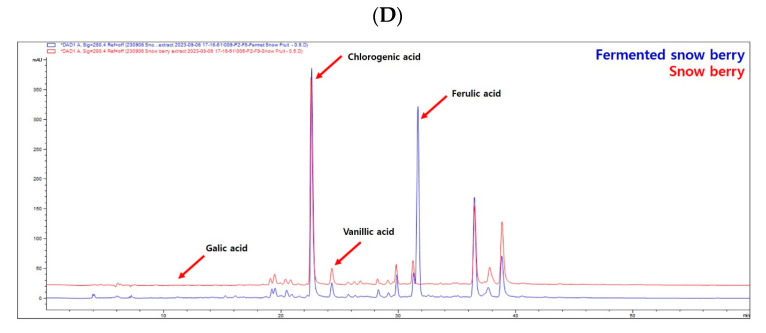
HPLC analysis of each part of the snowberry before and after fermentation was performed to identify and quantify the main constituents. (**A**) Representative HPLC chromatogram of Snowberry Root Extract (SRE) and Fermented Snowberry Root Extract (FSRE). (**B**) Representative HPLC chromatogram of Snowberry Stem Extract (SSE) and Fermented Snowberry Stem Extract (FSSE). (**C**) Representative HPLC chromatograms of Snowberry Leaf Extracts (SLEs) and Fermented Snowberry Leaf Extract (FSLE). (**D**) Representative HPLC chromatogram of Snowberry Fruit Extract (SFE) and Fermented Snowberry Fruit Extract (FSFE).

Standards of gallic acid, chlorogenic acid, vanillic acid, and ferulic acid (Sigma-Aldrich, St. Louis, MO, USA) were analysed using HPLC at different concentrations to create calibration curves. Snowberry Extracts from each fermentation stage were analysed by HPLC. Based on the calibration curves, we identified and quantified the components of each Snowberry Extract. Gallic, chlorogenic, vanillic, and ferulic acids were present in all extracts (Table 1 and Figure 1). 

Chlorogenic acid content increased in Fermented Snowberry Fruit Extract (FSFE), and ferulic acid, which was not detected before fermentation, increased significantly in FSFE. In Fermented Snowberry Leaf Extract (FSLE), chlorogenic acid decreased while ferulic acid increased significantly. The Fermented Snowberry Root Extract (FSRE) showed a decrease in vanillic acid and an increase in ferulic acid levels. The FSSE showed a decrease in vanillic acid levels. Gallic acid content did not significantly differ before and after fermentation (Table 2 and Figure 1).

### 2.2. Evaluation of the Moisturising Properties of the SE and FSE

**Figure 2:**
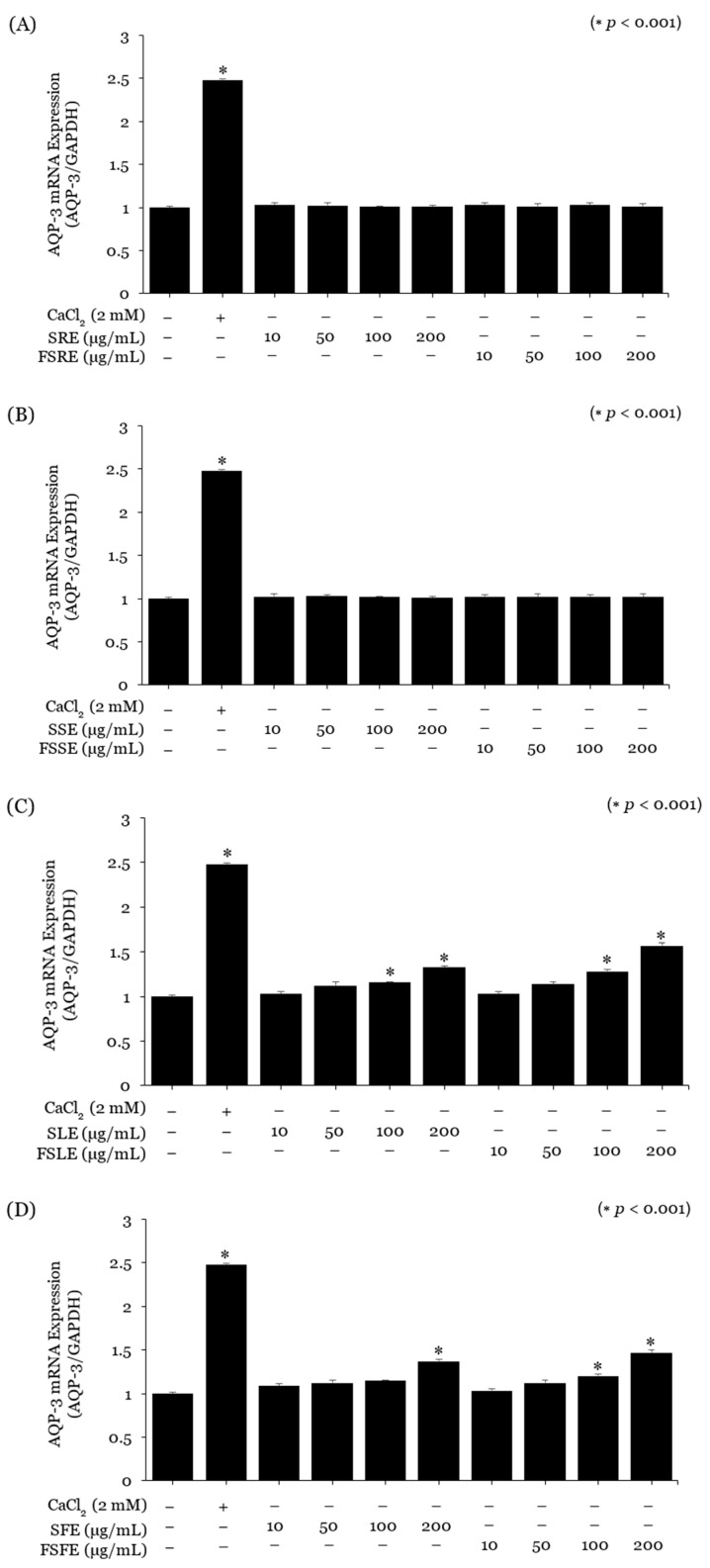
Effect of the SE and FSE on skin hydration function. The expression levels of the skin hydration-related gene aquaporin 3 (AQP-3) were measured by real-time PCR in keratinocytes. (**A**) Efficacy of Snowberry Root Extract (SRE) and Fermented Snowberry Root Extract (FSRE). (**B**) Efficacy of Snowberry Stem Extract (SSE) and Fermented Snowberry Stem Extract (FSSE). (**C**) Efficacy of Snowberry Leaf Extract (SLE) and Fermented Snowberry Leaf Extract (FSLE). (**D**) Efficacy of Snowberry Fruit Extract (SFE) and Fermented Snowberry Fruit Extract (FSFE). CaCl_2_ (2 mM) was used as a positive control. Values represent the mean ± standard deviation (SD) of three independent experiments, and statistically significant values are indicated (* *p* < 0.001, compared with the housekeeping gene GAPDH).

**Figure 3:**
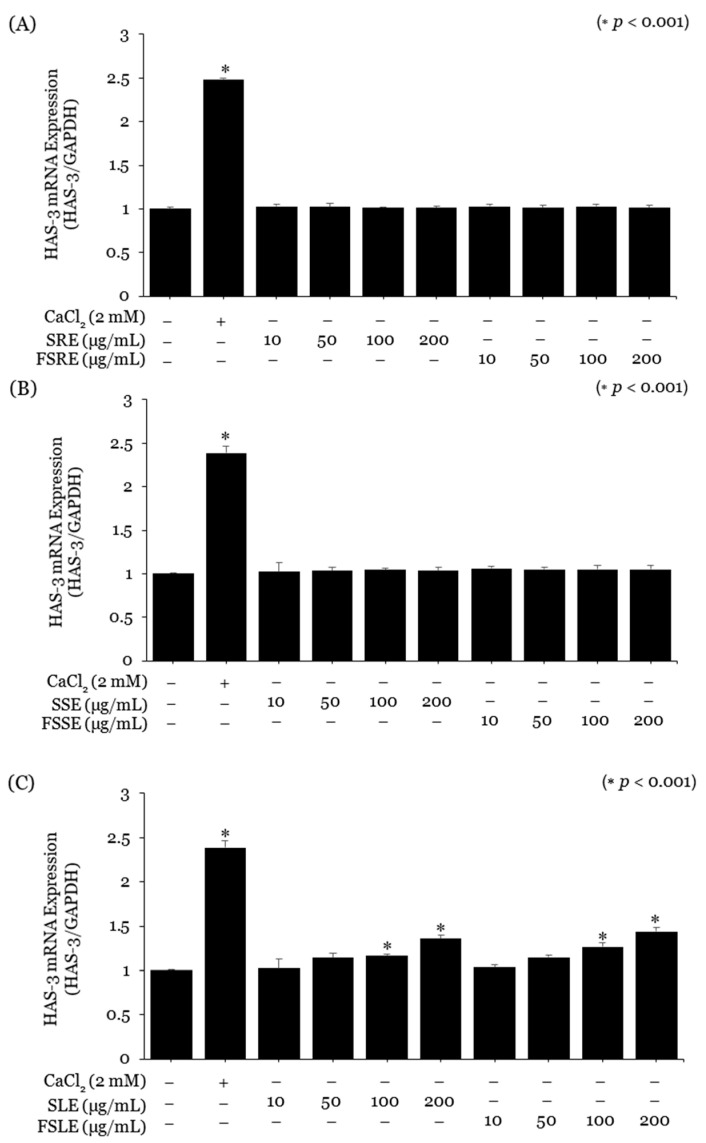

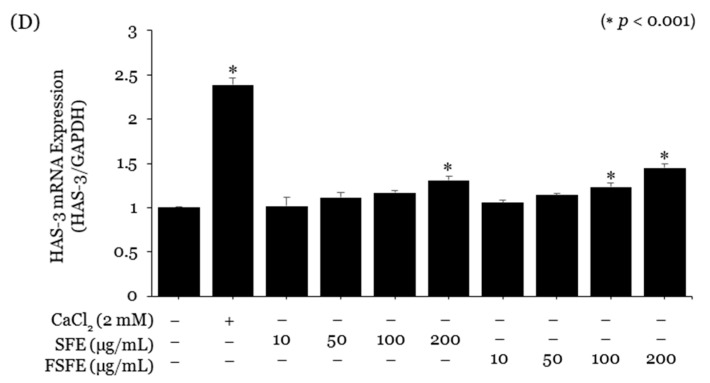
Effect of the SE and FSE on skin hydration function. The expression levels of the hyaluronic acid synthesis-related gene hyaluronan synthase 3 (HAS-3) were measured by real-time PCR in keratinocytes. (**A**) Efficacy of Snowberry Root Extract (SRE) and Fermented Snowberry Root Extract (FSRE). (**B**) Efficacy of Snowberry Stem Extract (SSE) and Fermented Snowberry Stem Extract (FSSE). (**C**) Efficacy of Snowberry Leaf Extract (SLE) and Fermented Snowberry Leaf Extract (FSLE). (**D**) Efficacy of Snowberry Fruit Extract (SFE) and Fermented Snowberry Fruit Extract (FSFE). CaCl_2_ (2 mM) was used as the positive control. Values represent the mean ± standard deviation (SD) of three independent experiments, and statistically significant values are indicated (* *p* < 0.001, compared with the housekeeping gene GAPDH).

Keratinocytes were treated with non-toxic concentrations of snowberry, and fermented extracts evaluated their moisturising effects. The Snowberry Root Extract (SRE), Stem Extract (SSE), FSRE, and FSSE did not significantly affect the mRNA expression of AQP-3 or HAS-3 (Figure 2A,B and Figure 3A,B). However, SLE increased AQP-3 gene expression approximately 1.12-fold at 50 μg/mL and 1.32-fold at 200 μg/mL. FSLE showed the highest moisturising effect, increasing AQP-3 expression approximately 1.57-fold at 200 μg/mL (Figure 2C). Similarly, HAS-3 gene expression increased in a concentration-dependent manner with SLE and FSLE starting at 50 μg/mL. The SLE increased HAS-3 expression approximately 1.36-fold at 200 μg/mL, while the FSLE increased it approximately 1.44-fold at 200 μg/mL (Figure 3C). In fruit extracts, the SFE increased AQP-3 gene expression approximately 1.37-fold at 200 μg/mL, and the FSFE increased it approximately 1.47-fold at 200 μg/mL (Figure 2D). HAS-3 expression also showed a concentration-dependent increase, with the SFE increasing expression approximately 1.31-fold at 200 μg/mL and the FSFE increasing it approximately 1.45-fold at 200 μg/mL (Figure 3D).

### 2.3. Evaluation of the SE and FSE for Wrinkle Treatment

**Figure 4:**
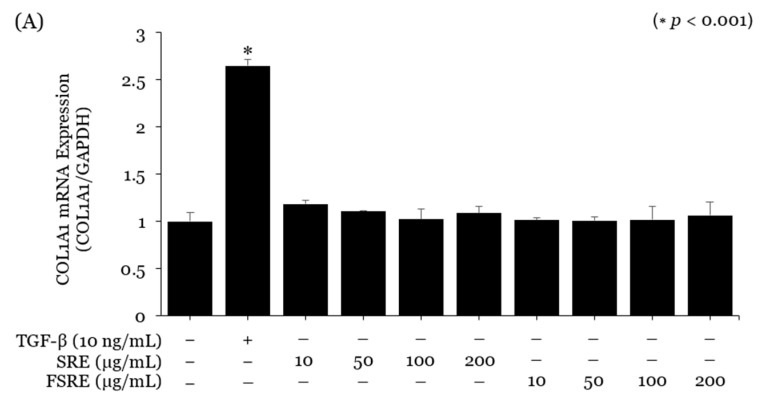

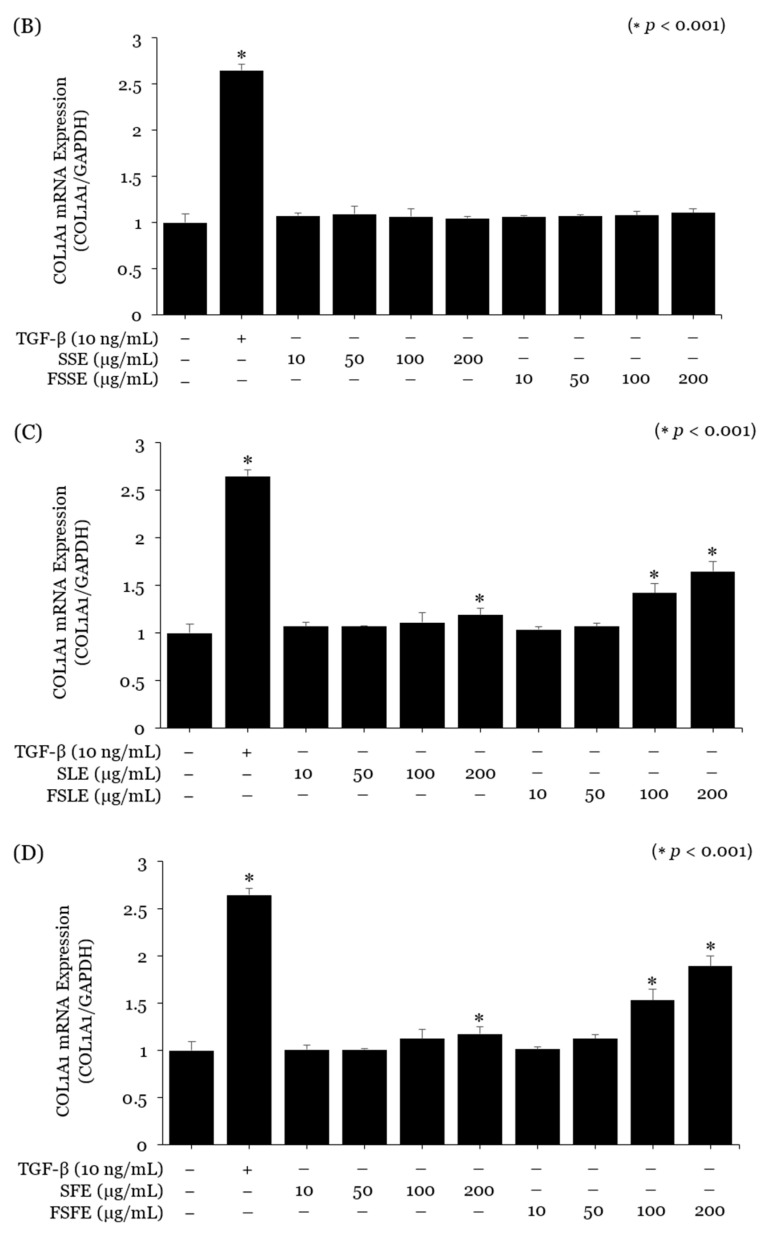
Effect of the SE and FSE on anti-ageing function. The expression levels of the collagen synthesis gene collagen type I alpha 1 (COL1A1) were measured by real-time PCR in dermal fibroblasts. (**A**) Efficacy of Snowberry Root Extract (SRE) and Fermented Snowberry Root Extract (FSRE). (**B**) Efficacy of Snowberry Stem Extract (SSE) and Fermented Snowberry Stem Extract (FSSE). (**C**) Efficacy of Snowberry Leaf Extract (SLE) and Fermented Snowberry Leaf Extract (FSLE). (**D**) Efficacy of Snowberry Fruit Extract (SFE) and Fermented Snowberry Fruit Extract (FSFE). TGF-β (10 ng/mL) was used as a positive control. Values represent the mean ± standard deviation (SD) of three independent experiments, and statistically significant values are indicated (* *p* < 0.001, compared with the housekeeping gene GAPDH).

To evaluate the anti-wrinkle effects of snowberry and fermented extracts, fibroblasts were treated with non-toxic concentrations of the extracts. The results showed that the SRE, SSE, FSRE, and FSSE did not significantly alter COL1A1 mRNA expression. However, the SLE increased COL1A1 gene expression approximately 1.11-fold at 100 μg/mL and 1.19-fold at 200 μg/mL. The FSLE showed the highest COL1A1 expression, increasing approximately 1.65-fold at 200 μg/mL. In fruit extracts, the SFE increased COL1A1 gene expression approximately 1.18-fold at 200 μg/mL, while the FSFE increased it approximately 1.89-fold at 200 μg/mL (Figure 4).

## 3. Discussion

This study investigated the potential cosmetic benefits of snowberry and its fermented extracts, focusing on previously unexplored plant parts. Our results revealed significant variations in phenolic acid content, particularly ferulic acid, which was not present in the non-fermented leaf and fruit extracts but was detected at notable levels after fermentation. This increase in ferulic acid may play a key role in the observed upregulation of AQP-3, HAS-3, and COL1A1 gene expression, suggesting a possible link between fermentation and enhanced skin benefits. The increase in ferulic acid levels in the fermented extracts is of particular interest, given its known biological activities. Ferulic acid is a well-documented antioxidant that can mitigate oxidative stress induced by UV radiation. Previous studies have shown that ferulic acid can inhibit the expression of MMP-2 and MMP-9, enzymes associated with collagen degradation, which are typically upregulated following UVB exposure [22]. This suggests that the increase in ferulic acid following fermentation could contribute to the protective effects against UV-induced skin damage observed in this study. Furthermore, ferulic acid has been reported to promote wound healing and support angiogenesis, particularly in diabetic models [23,24]. These properties are essential in maintaining skin integrity and could explain the enhanced skin benefits observed with the fermented extracts. The significant upregulation of AQP-3 and HAS-3 gene expression in response to fermented leaf and fruit extracts suggests that these extracts may enhance skin hydration. AQP-3 is critical for maintaining skin moisture by facilitating water transport into epidermal cells, while HAS-3 is involved in the synthesis of hyaluronic acid, a key component in skin hydration and elasticity. The observed upregulation of these genes aligns with the increased ferulic acid content, which might stimulate their expression, thereby enhancing skin moisturization. Similarly, the significant increase in COL1A1 gene expression observed with the fermented extracts, particularly from the leaves and fruits, points to their potential anti-wrinkle effects. COL1A1 is a major component of the dermal extracellular matrix, and its expression is essential for maintaining skin structure and preventing wrinkle formation. The increase in COL1A1 expression in fermented extracts could be attributed to the presence of ferulic acid, which has been shown to protect dermal collagen from degradation and promote collagen synthesis [25].

Overall, these findings suggest that fermentation enhances the bioavailability and efficacy of key phenolic compounds in snowberry, particularly ferulic acid, which in turn may contribute to the observed moisturising and anti-wrinkle effects. This study provides novel insights into the potential skin benefits of snowberries, highlighting the importance of fermentation in enhancing their bioactive properties.

These results underscore the potential of fermentation as a valuable process in enhancing the beneficial effects of natural extracts, particularly in the context of skincare. Further research is warranted to explore the underlying mechanisms through which fermentation increases the levels of bioactive compounds like ferulic acid and to determine the full scope of their impact on skin health. Such investigations could ultimately contribute to the development of more advanced and effective skincare products. 

## 4. Materials and Methods

### 4.1. Preparation of SE Products

The *Symphoricarpos albus (snowberry)* roots, stems, leaves, and fruits were cultivated and verified for species compatibility by YP Jang, one of the authors, who is a professor of traditional Korean medicine and holds a Ph.D. in pharmacy. The snowberries were harvested from summer to autumn, cut into approximately 1 cm pieces immediately after harvest, dried, and stored in a −70 °C deep freezer for further use. Primary extraction was performed on each plant part (roots, stems, leaves, and fruits) using ultrasonic extraction (JeioTech, Seoul, South Korea; frequency: 40 kHz; power: 200 W) with 10 volumes of 70% ethanol for 2 h, followed by 24 h immersion extraction and primary filtration. A secondary extraction was conducted with 10 volumes of 50% ethanol using the same ultrasonic extraction method, followed by a 24 h immersion extraction and secondary filtration. Once the extraction process was completed, the extracts were subjected to sterile filtration using 0.45 μm and 0.2 μm filters. The root, stem, and fruit extracts were concentrated and lyophilised using a rotatory evaporator (Eyela, Tokyo, Japan) and freezer dryer (Eyela, Japan). The leaf extract was treated with hexane to remove chlorophyll, then concentrated and lyophilised. The resulting powders were dissolved in methanol, sonicated, and filtrated through a 0.45 μm membrane filter. The extracts were designated as SRE, SSE, leaf extract (SLE), and fruit extract (SFE).

### 4.2. Preparation of FSE Products

*Lactobacillus plantarum* KCTC 14687BP, isolated from *Centella asiatica*, was used for snowberry fermentation. While the strain was originally isolated from a medicinal plant, the MRS medium for industrial fermentation was modified to suit the fermentation process. The medium was prepared in 500 mL batches, autoclaved, and then supplemented with 1% (*w*/*v*) freeze-dried Snowberry Extract (as detailed in Table 3). After two subcultures, 1% (*w*/*v*) *Lactobacillus plantarum* was inoculated into MRS medium containing Snowberry Extracts from various plant parts and incubated at 37 °C for 24 h under stationary conditions. Upon completion of the incubation, microbial contamination and fermentation success were assessed using various agar plates, including Nutrient Agar, Potato Dextrose Agar, MacConkey Agar, and BCP Count Agar. The fermentation conditions were optimised through preliminary experiments by assessing microbial growth conditions and post-fermentation component analysis. The fermented extracts were designated as FSRE, FSSE, FSLE, and FSFE. All experiments and analyses were performed in triplicate.

### 4.3. HPLC Analysis of the SE and FSE

HPLC analysis was conducted to identify and quantify the major components of each extract. The chromatographic instrument used in this experiment was an Agilent 1290 Infinity II HPLC system (Aglient Technology Inc., Santa Clara, CA, USA) with a Capcell pak C18 4.6 mm × 250 mm, 5 µm column (Shiseido Co, Osaka, Japan). The mobile phases were analysed under gradient solvent conditions using 0.2% acetic acid (Sigma-Aldrich, St. Louis, MO, USA) and acetonitrile (J.T. Baker, Phillipsburg, NJ, USA) with 0.2% acetic acid. All solvents were degassed and filtered before use. The column flow rate and temperature were set at 1.0 mL/min and 30 °C, respectively. The sample injection volume was 10 µL, and the HPLC/UV was measured at 280 nm (Table 4). Reference chemicals, including gallic acid, chlorogenic acid, vanillic acid, and ferulic acid, were purchased from Sigma-Aldrich (St. Louis, MO, USA).

### 4.4. Real-Time Polymerase Chain Reaction (RT-PCR)

Neonatal human epidermal keratinocytes (Lonza, Walkersville, MD, USA) and human dermal fibroblasts (ATCC, Manassas, VA, USA) were cultured in T75 flasks at 37 °C and 5% CO_2_ in their respective media. Cells were seeded at approximately 5 × 10^5^ cells/mL in 12-well plates, stabilised, and starved in serum-free DMEM. Cells were treated with Snowberry Extract and Fermented Snowberry Extract at concentrations of 10, 50, 100, and 200 μg/mL for 24 h. RNA was extracted using an RNA Extraction Kit (Macherey-Nagel, Dueren, Germany) and converted to cDNA with a cDNA Synthesis Kit (Toyobo, Osaka, Japan). cDNA purity and concentration were assessed using a NanoDrop, then diluted and subjected to real-time PCR with Universal SYBR Green Supermix (Bio-Rad, Hercules, CA, USA). The analysis was conducted using the CFX Connect system (BIO-RAD, USA), and the expression levels of the AQP-3, HAS-3, and COL1A1 genes were normalised against the housekeeping gene GAPDH. For the positive control, 2 mM CaCl2 (Sigma-Aldrich, St. Louis, MO, USA) and 10 ng/mL TGF-β (Sigma-Aldrich, St. Louis, MO, USA) were used. The normalised gene expression levels were compared with the untreated control group to assess gene expression levels. The information on the primers used in the experiment is presented in Table 5.

### 4.5. Statistical Analysis

All experiments were performed in triplicate. Statistical analysis was performed using one-way analysis of variance, followed by Dunnett’s test and Student’s *t*-test between control and treated groups. Data are presented as mean ± standard deviation (SD), with *p* < 0.001 considered statistically significant (* *p* < 0.001).

## 5. Conclusions

In conclusion, this study demonstrates that both the leaf and fruit extracts of snowberries, as well as their fermented counterparts, have the potential to enhance skin hydration and reduce wrinkles. These findings pave the way for developing novel cosmetic ingredients derived from snowberries, offering natural and effective solutions for skincare.

## Figures and Tables

**Table 1:** Phytochemical analysis of Snowberry Extracts from each part by HPLC.

(Units: mg/g)	Gallic Acid	Chlorogenic Acid	Vanillic Acid	Ferulic Acid
Stem	0.07 ± 0.004	16.97 ± 0.43	4.10 ± 0.21	0.15 ± 0.012
Leaf	0.10 ± 0.007	41.98 ± 0.71	2.88 ± 0.19	0.00
Root	0.13 ± 0.01	15.02 ± 0.0.57	2.26 ± 0.06	0.26 ± 0.034
Fruit	0.00	25.43 ± 0.83	1.50 ± 0.09	0.00

**Table 2:** Phytochemical analysis of Fermented Snowberry Extracts from each part by HPLC.

(Units: mg/g)	Gallic Acid	Chlorogenic Acid	Vanillic Acid	Ferulic Acid
Stem	0.10 ± 0.014	16.18 ± 0.31	0.97 ± 0.072	0.21 ± 0.012
Leaf	0.10 ± 0.025	38.21 ± 0.92	1.58 ± 0.049	11.45 ± 0.16
Root	0.14 ± 0.019	14.61 ± 0.45	2.35 ± 0.067	0.42 ± 0.037
Fruit	0.00	28.01 ± 0.71	0.86 ± 0.019	13.21 ± 0.092

**Table 3:** Composition of the *Lactobacillus* fermentation medium.

Components	Ratio of Contents (%)
Glucose	1%
Soy peptone (soy protein)	1%
Yeast extract	0.5%
Sodium acetate	0.25%
Dipotassium phosphate	0.1%
Ammonium citrate	0.1%
Tween 80	0.05%
Magnesium sulphate	0.005%
Manganese sulphate	0.0025%
Lyophilised snowberry extract powder	1%

**Table 4:** Conditions for the HPLC analysis.

Column	Capcell pak C18 4.6 mm × 250 mm, 5 µL	
Flow rate	1.0 mL/min	
Detection	UV 280 nm	
Temperature	30 °C	
Injection vol	10 μL	
Mobile phase (min)	0.2% Acetic acid in water	0.2% Acetic acid in ACN
0	95	5
30	70	30
55	65	35
58	95	5
60	95	5

**Table 5:** Real-time PCR primer sequences.

Target Gene	Forward Primer	Reverse Primer
GAPDH	ACCACAGTCCATGCCATCAC	TCCACCACCCTGTTGCTGTA
COL1A1	AGGGCCAAGACGAAGACATC	AGATCACGTCATCGCACAACA
HAS-3	CCCAGCCAGATTTGTTGATG	AGTGGTCACGGGTTTCTTCC
AQP3	AGACAGCCCCTTCAGGATTT	TCCCTTGCCCTGAATATCTG

## Data Availability

No publicly archived datasets are available for this work.
